# Adversity in Childhood and Measures of Aging in Midlife: Findings From a Cohort of British Women

**DOI:** 10.1037/pag0000182

**Published:** 2017-09

**Authors:** Emma L. Anderson, Jon Heron, Yoav Ben-Shlomo, Diana Kuh, Rachel Cooper, Debbie A. Lawlor, Abigail Fraser, Laura D. Howe

**Affiliations:** 1 Medical Research Council Integrative Epidemiology Unit, and School of Social and Community Medicine, University of Bristol; 2 School of Social and Community Medicine, University of Bristol; 3 Medical Research Council Unit for Lifelong Health and Ageing, University College London; 4 Medical Research Council Integrative Epidemiology Unit, and school of Social and Community Medicine, University of Bristol; 5 School of Social and Community Medicine, University of Bristol

**Keywords:** childhood, adversity, socioeconomic, cognitive, physical

## Abstract

Very few studies have assessed whether socioeconomic and psychosocial adversity during childhood are associated with objective measures of aging later in life. We assessed associations of socioeconomic position (SEP) and total psychosocial adversity during childhood, with objectively measured cognitive and physical capability in women during midlife. Adverse childhood experiences were retrospectively reported at mean ages 28–30 years in women from the Avon Longitudinal Study of Parents And Children (*N* = 2,221). We investigated associations of childhood SEP and total psychosocial adversity, with composite measures of cognitive and physical capability at mean age 51 years. There was evidence that, compared with participants whose fathers had professional occupations, participants whose fathers had managerial/technical, skilled nonmanual, skilled manual, and partly or unskilled manual occupations had, on average, lower physical and cognitive capability. There was a clear trend for increasing magnitudes of association with lowering childhood SEP. There was also evidence that greater total psychosocial adversity in childhood was associated with lower physical capability. Total psychosocial adversity in childhood was not associated with cognitive capability. Lower SEP in childhood is detrimental to cognitive and physical capability in midlife, at least in part, independently of subsequent SEP in adulthood. Greater psychosocial adversity in childhood is associated with poorer physical capability, independently of social disadvantage in childhood. Our findings highlight the need for interventions to both identify and support children experiencing socioeconomic or psychosocial of adversity as early as possible.

Maintaining physical and cognitive capabilities in older age is essential for functional independence (Reed et al., 1998), and lower levels of cognitive and physical capability, even in midlife, are associated with higher rates of all-cause mortality (Cooper et al., 2010). Thus, determining factors that are associated with poorer cognitive and physical capability is important and may reduce the associated economic (Callahan, Hendrie, & Tierney, 1995) and care burden (Garand, Dew, Eazor, DeKosky, & Reynolds, 2005). Studies have previously reported both socioeconomic (e.g., low head of household social class, parental education) and psychosocial (e.g., sexual or physical abuse) adversity to be associated with lower cognitive and physical capability later in life (Birnie et al., 2011; Fors, Lennartsson, & Lundberg, 2009; Lyu & Burr, 2016; Montez & Hayward, 2014; Richards & Wadsworth, 2004; Schussler-Fiorenza Rose, Xie, & Stineman, 2014; Surtees & Wainwright, 2007; Turrell et al., 2002). There are several plausible mechanisms through which these associations could occur, including psychological (e.g., through greater risk of stress, anxiety and depression [Ege, Messias, Thapa, & Krain, 2015]), behavioral (e.g., through increased smoking or alcohol consumption [Dube, Anda, Felitti, Edwards, & Croft, 2002]) and biological (e.g., through higher levels of stress hormones and systemic inflammation [Danese & McEwen, 2012]).

The association between childhood socioeconomic position (SEP) and cognitive capability in adulthood is now well established (Dugravot et al., 2009; Fors et al., 2009; Horvat et al., 2014; Kaplan et al., 2001; Lyu & Burr, 2016; Marengoni, Fratiglioni, Bandinelli, & Ferrucci, 2011). However, much less evidence exists for the impact of childhood SEP on objective (as opposed to self-report) measures of physical capability (Birnie et al., 2011; Guralnik & Ferrucci, 2003). Very few studies have examined whether psychosocial adversity carries additional risks for later cognitive and physical health, over and above socioeconomic disadvantage. Furthermore, few studies have assessed whether these associations are mediated by SEP in adulthood (i.e., whether psychosocial adversity in childhood increases risk of lower SEP in adulthood, e.g., by reducing self-confidence and the ability to achieve in school or in later employment), which in turn reduce later cognitive and physical capability. Examining potential mediators may help to highlight possible targets for interventions.

Existing studies assessing associations of psychosocial adversity in childhood and later cognitive and physical health have either not considered possible confounding by childhood SEP or have focused on single adverse experiences such as sexual abuse or physical abuse, without considering a possible total effect of multiple adverse experiences (Feeney, Kamiya, Robertson, & Kenny, 2013). Assessing total psychosocial adversity acknowledges that adverse experiences tend to co-occur and that experiencing multiple forms of adversity may have a greater adverse effect on physical and cognitive aging than experiencing only one. Very few studies have considered the co-occurrence of multiple forms of psychosocial and/or socioeconomic adversity in relation to impaired cognition in adulthood, and those few existing studies have all used a simple summary adversity scores (i.e., totaling the number of adverse experiences) (Anda et al., 2006; Lovallo et al., 2013; Reuben et al., 2016). This method has important limitations (Howe, Tilling, & Lawlor, 2015) because it assumes that each adverse experience has the same direction and magnitude of association with the outcome.

In this study, we aimed to investigate associations of retrospectively reported childhood SEP and psychosocial adversity (both total and individually by maternal lack of care, maternal overprotection, maladaptive family functioning, parental mental illness, sexual abuse and physical or emotional cruelty or neglect) with cognitive and physical capability in midlife. We examined (a) whether psychosocial adversity is associated with cognitive and physical capability, over and above childhood SEP; (b) whether any observed associations are mediated by adult SEP; and (c) whether associations of psychosocial adversity with cognitive and physical capability differ in women with high, compared with low, childhood SEP and in women with high, compared with low, adult SEP.

## Method

### Study Population

**Figure 1 fig1:**
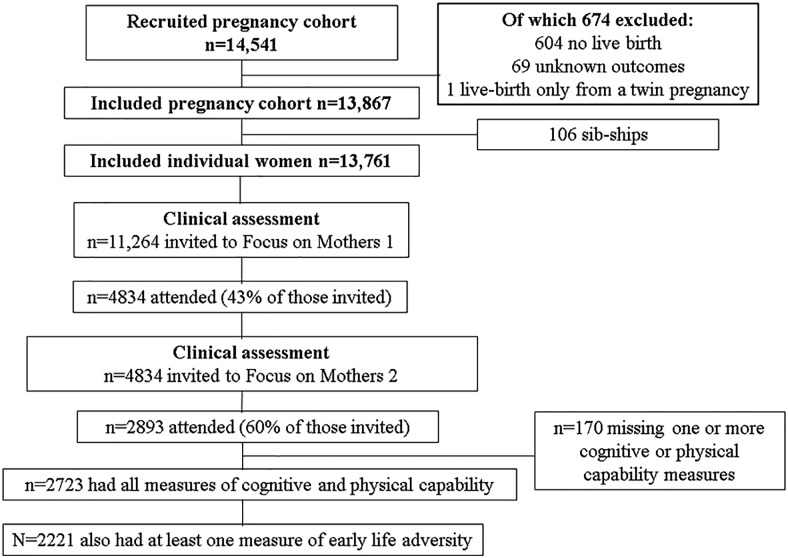
Participant flow through the study.

The Avon Longitudinal Study of Parents and Children (ALSPAC) is a prospective birth cohort study from southwest England that recruited 14,541 pregnant women resident in three Bristol-based health districts, with an expected date of delivery between April 1991 and December 1992. Our analysis uses data from the mothers in this cohort (Fraser et al., 2013). The study web site contains details of all available data through a fully searchable data dictionary (www.bris.ac.uk/alspac/researchers/data-access/data-dictionary). Ethical approval for the study was obtained from the ALSPAC Ethics and Law Committee and the local research ethics committees. Approximately 25 years after recruitment into the cohort, women were invited to attend a follow-up research clinic at which cognitive and physical capability was assessed. A total of 2,893 women attended this clinic (mean age 51 years, *SD* 4.4 years). Eligible participants had data for paternal occupational social class, responded to at least one question about psychosocial adversity in childhood, and had data for all measures of cognitive and physical capability (*n* = 2221). One hundred seventy women were excluded because of missing one or more cognitive or physical capability measures (see Figure 1).[Fig-anchor fig1]

### Assessing SEP and Psychosocial Adversity in Childhood

#### Psychosocial adversity

Women retrospectively reported childhood psychosocial adversity in questionnaires administered at the time of enrollment into the study, throughout pregnancy, and postnatally (from 12 weeks’ gestation to 33 months postnatally, mean ages at the time of reporting ranged between 28 and 30 years). A priori, we aimed to examine the same adversity measures as the Adverse Childhood Experiences study. However, ALSPAC measured many additional forms of adversity to this study. Thus, we decided to include as many types of psychosocial adversity as possible.

The following forms of psychosocial adversity were assessed in the questionnaires: maternal lack of care and maternal overprotection, maladaptive family functioning, parental mental illness, sexual abuse, and nonsexual abuse. Questions about maternal care and overprotection were based on a validated instrument for assessing maternal bonding (Parker, 1990). Maladaptive family functioning includes questions that assess the nature of the relationship between the participant’s mother and father (i.e., whether the relationship was, e.g., stable and predictable, affectionate, violent, respectful). Parental mental illness includes questions about depression, anxiety, schizophrenia, or alcoholism in the participant’s mother or father. Sexual abuse questions assessed experiences of various types of sexual abuse by different people (e.g., family members, friends or strangers). Nonsexual abuse includes questions that capture physical or emotional cruelty and neglect by either parent/guardian. It is important to note that although there may appear to be overlap between maternal lack of care and emotional cruelty or neglect, the questions assessing the latter reflect neglect by either parent/guardian, not just the mother. Details of the exact questions asked about each type of psychosocial adversity are provided in the online supplement.

#### Childhood SEP

At enrollment into the study, women retrospectively reported their mother’s and father’s occupation during their childhood. Missing data were much higher for the mother’s occupation than for the father’s, likely because of the high proportion of women who did not work outside the home during that period. Thus, we decided to use the father’s occupation where this information was available and use the mother’s occupation only when the father’s was not reported and the mother’s was. The father’s occupation was coded as professional, managerial and technical, skilled nonmanual, skilled manual, and partly or unskilled manual occupations, in line with the Standard Occupational Classification, 2000.

### Assessing Cognitive and Physical Capability in Midlife

Cognitive and physical capability outcomes were assed at a follow-up research clinic approximately 23 years after the assessment of childhood SEP and psychosocial adversity. All cognitive and physical capability outcomes measured in this study are associated with mortality (Cooper et al., 2010; Cooper, Strand, Hardy, Patel, & Kuh, 2014; Small & Bäckman, 1997). Physical capability was assessed with a height-adjusted grip strength test, a timed chair rise, a timed one-leg standing balance test with eyes closed, and a 3-m timed walk test. Cognitive capability was assessed with verbal fluency (Lezak, 2004), logical memory,(Wechsler, 1998b), delayed logical memory (Wechsler, 1998b), digit backward (Wechsler, 1998a), digit symbol coding (Wechsler, 1998a), and spot-the-word tests (Baddeley, Emslie, & Nimmo-Smith, 1993). Full assessment details of each cognitive and physical capability test is provided in the online supplement.

### Covariables

Participants’ SEP in adulthood was reported at enrollment into the study (during years 1991–1992) as the highest of own and partner’s occupational class groups using the 1991 British Office of Population and Census Statistics classification. It was coded as professional, managerial and technical, skilled nonmanual, skilled manual, and partly or unskilled manual. Women reported their ethnicity in questionnaires administered at enrollment. Age at the time of outcome assessment was recorded.

## Statistical Analysis

### Generating Composite Scores of Cognitive and Physical Capability

In addition to assessing individual cognitive and physical capability tests, which reflect different underlying systems (e.g., fluid vs. crystallized intelligence, physical strength vs. balance), composite scores of cognitive and physical capability were also created using the method devised by Guralnik et al (Guralnik, Butterworth, Wadsworth, & Kuh, 2006). Combining measures into a composite score may identify a much higher risk group (i.e., participants doing very badly on all tests), thus allowing us to assess the extremes of physical and cognitive performance, which may be more revealing in a middle-aged population that is generally functioning well. Grip strength was adjusted for body size by dividing it by height. Each cognitive and physical capability test score was rescaled to lie between 0 and 1, giving all measures equal weight in the final composite scores (see online supplement for further details of the rescaling procedure). Chair rise speed and 3-m timed walk scores were reversed so that all scores were coded in the same direction, with 0 reflecting *poorest* and 1 reflecting *highest performance*. Participants unable to perform a test were assigned a value of 0. Rescaled cognitive and physical capability measures were summed to create normally distributed aggregate cognitive and physical capability scores, with ranges of 0–4 and 0–6, respectively.

### Total Psychosocial Adversity in Childhood

Most existing studies that have assessed total psychosocial adversity in childhood have used simple summary scores (i.e., totaling the number of adverse experiences; Crowell et al., 2016; Halonen et al., 2015; Su et al., 2015). Summary scores, arguably unrealistically, assume each adverse exposure to have the same direction and magnitude of association with the outcome. We used a data-driven approach to create a total psychosocial adversity score that weights each adversity exposure based on how strongly it correlates with other adversity exposures (i.e., allocating exposures that tend to co-occur with others a higher weight so that they contribute more to the total adversity score).

**Figure 2 fig2:**
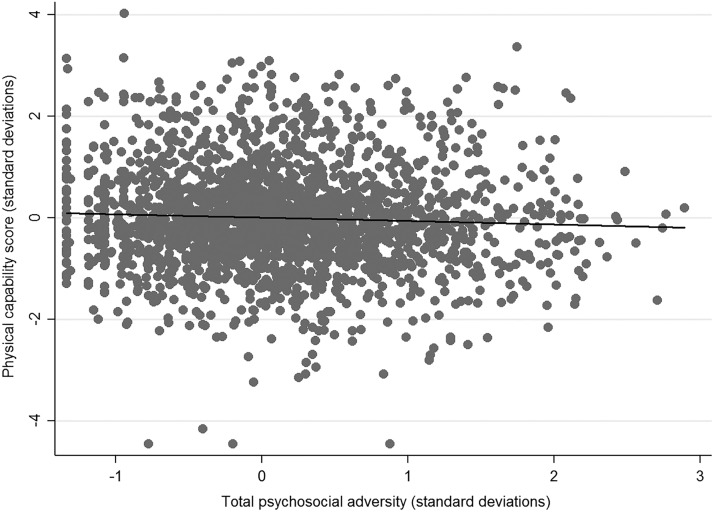
Scatter plot and regression line of standardized physical capability scores by standardized levels of total psychosocial adversity (*n* = 2,221). More psychosocial adversity in childhood was associated with lower physical capability in midlife.

Because there were multiple questions assessing each specific type of adversity, we first sought to combine all available questions into single variables. Thus, we used confirmatory factor analysis (Tucker & Lewis, 1973) to create single latent constructs for maternal lack of care, maternal overprotection, parental mental illness, household dysfunction, sexual abuse, and nonsexual abuse (Figure 2 and Supplemental Table 1). We then estimated a latent construct of total psychosocial adversity in childhood, which was informed by each of these single latent constructs. Latent constructs are variables that are not directly observed but are inferred from other variables that are observed or measured (i.e., responses to the adversity questions). Higher latent trait values are indicative of greater levels of adversity. Full methods and model fit statistics for the confirmatory factor analyses are provided in the online supplement. Analyses were conducted using Mplus version 7.31 (Muthén & Muthén, 1998–2010).[Fig-anchor fig2]

Structural equation models (Supplemental Figure 1 of the online supplement) were used to simultaneously conduct the factor analyses and estimate associations of total psychosocial adversity in childhood with cognitive and physical capability, in the following regression models: (a) unadjusted; (b) adjusted for age at outcome assessment and ethnicity; (c) additionally adjusted for concurrent forms of adversity (i.e., associations of childhood psychosocial adversity are adjusted for SEP and vice versa); and (d) additionally adjusted for potential mediation by adult SEP.

### Missing Data and Additional Analyses

Our main analysis dealt with missing data using the weighted least squares means and variance adjusted estimator, which permits the inclusion of women with incomplete data, assuming data are missing at random, conditional all other exogenous variables in the model (Edwards, Holden, Felitti, & Anda, 2003). As a sensitivity analysis, we repeated analysis in the sample with no missing data for any variable. We also assessed associations of each specific form of psychosocial adversity with cognitive and physical capability. We examined whether associations between total psychosocial adversity and the outcomes differ in (a) women who have a high (professional, managerial, and technical occupations) childhood SEP compared with low (skilled, partly skilled, and unskilled occupations), and (b) women who have a high (professional, managerial, and technical occupations) adult SEP compared with low (skilled, partly skilled, and unskilled occupations). We used binary childhood and adulthood SEP variables to assess these interactions because we do not have a large-enough sample size (and thus statistical power) to investigate interactions between the five different SEP categories used for the main analyses. We examined associations of SEP and total psychosocial adversity with each individual cognitive and physical capability measure. Finally, we compared findings from our main analyses with those in which we used a more traditional approach of assessing total psychosocial adversity, a simple additive score. The additive score was created for participants with complete data for all the adversity measures and physical and cognitive outcome data (*n* = 1,535). Full details of the additive score are in the online supplement (Supplemental Table 2).

## Results

There was evidence that women included in these analysis, on average, had a higher 3-m timed walk speed and higher cognitive capability scores, were more likely to be white, and have a higher SEP compared with women excluded because of missing data (see Table 1). However, the magnitude of the differences was small. Correlations between each of the cognitive and physical capability measures (Supplemental Tables 3 and 4) were weak to moderate: Pearson’s *r* ranges were 0.07–0.25, and were 0.15–0.41 for cognitive and physical capability measures, respectively. Logical memory and delated logical memory were strongly correlated (*r* = .84). Women with a low childhood SEP were more likely to have experienced physical neglect, emotional neglect, parental separation or absence, and a dysfunctional household compared with women with a high childhood SEP (Supplemental Table 5). Of women who had a low childhood SEP, 23% went on to have a high adulthood SEP. Of women with a high childhood SEP, 58% went on to have a low adulthood SEP.[Table-anchor tbl1]

### Associations of Childhood SEP With Cognitive and Physical Capability

There was evidence that, compared with participants whose fathers had professional occupations, participants whose fathers had managerial/technical, skilled nonmanual, skilled manual, and partly or unskilled manual occupations had, on average, lower physical (see Table 2) and cognitive (see Table 3) capability. There was evidence of increasing magnitudes of association with lowering childhood SEP, and associations remained, even after adjustment for potential confounding my age, ethnicity, and total psychosocial adversity in childhood and for potential mediation by adult SEP.[Table-anchor tbl2][Table-anchor tbl3]

### Associations of Total Psychosocial Adversity With Composite Cognitive and Physical Capability Scores

There was no evidence of an association between total psychosocial adversity and cognitive capability in any of the models (Supplemental Table 6 of the online supplement). There was evidence that greater total psychosocial adversity in childhood was associated with lower physical capability, after adjusting for age at outcome assessment and ethnicity (standardized β: −0.05, 95% confidence interval: −0.1 to 0.0004, *p* = .05, Figure 2, Supplemental Table 7 of the online supplement). The point estimate attenuated very little (from −0.05 to −0.04) after adjusting for potential confounding by SEP in childhood, but confidence intervals widened to include the null (*p* = .10). There was no evidence of an association after adjusting for potential mediation by adult SEP.

### Additional Analyses

There was evidence that having an overprotective or absent parent, being emotionally neglected, being adopted, or spending time in local authority care was associated with poorer cognitive capability in midlife. In contrast, having a physically ill parent was associated with better cognitive capability (Supplemental Table 8 of the online supplement). Parental lack of care or having a parent be physically cruel during childhood was associated with poorer physical capability. Low childhood SEP was associated with poorer scores for all individual cognitive capability measures (compared with high) and with poorer grip strength and standing balance (Supplemental Table 9). Greater total psychosocial adversity was associated with a slower 3-m timed walk and a lower digit symbol coding score. Associations of total psychosocial adversity with physical and cognitive capability were similar in women who had a high, compared with low, childhood SEP (interaction values of *p* > 0.1, Supplemental Table 10 of the online supplement) and in women with high compared with low adult SEP (interaction values of *p* > 0.1, Supplemental Table 11 in online supplement). Associations were similar in the sample with no missing data (Supplemental Table 12). Associations were very similar when using an additive score of psychosocial adversity rather than a latent construct, except that confidence intervals were slightly wider because of the reduction in sample size (*n* = 2221 in the main analysis of the latent construct compared with *n* = 1,535 in the additive score analysis, Supplemental Table 13 of the online supplement).

## Discussion

We found evidence that lower SEP in childhood is associated with poorer cognitive capability and objectively measured physical capability in midlife, at least in part independently of SEP in adulthood. We also found evidence that greater total psychosocial adversity in childhood is associated with poorer physical capability, independently of socioeconomic disadvantage in childhood. There was no evidence on an association between total psychosocial adversity and childhood SEP. There was no evidence that associations of total psychosocial adversity in childhood with cognitive and physical capability differed in participants with high, compared with low, childhood SEP or high, compared with low, SEP in adulthood. Overall, our findings imply that consequences of childhood SEP on both physical and cognitive capability and consequences of childhood psychosocial adversity on physical capability in women are likely to persist across the life course.

We did not observe that any particular type of psychosocial adversity was associated with cognitive or physical function more strongly than the other types. This potentially highlights that our study has insufficient power to detect associations with individual types of psychosocial adversity, particularly for those with low prevalences, such as sexual abuse. Importantly, our main analysis using an overall score of psychosocial adversity in childhood incorporates the widely recognized fact that different forms of psychosocial adversity often co-occur (Vachon, Krueger, Rogosch, & Cicchetti, 2015), and their effects may accumulate to influence cognitive and physical capability (Edwards et al., 2003).

In our study we assessed associations between childhood SEP and psychosocial adversity with each cognitive and physical capability test as well as the composite scores. Different cognitive and physical capability measures reflect different underlying systems (e.g., fluid vs. crystallized intelligence, physical strength vs. balance), and assessing them individually as opposed to using composite scores may help inform possible underlying pathways of association. Combining measures into a composite score may, however, increase power because summing them together identifies a much higher risk group (i.e., those performing very badly on all tests), which may drive associations. In our study, low childhood SEP (compared with high) was associated with poorer scores for all individual cognitive capability measures, suggesting that there is not one particular aspect of cognition that is largely affected by childhood SEP.

### Comparisons With Other Studies

Several studies have assessed associations of SEP in childhood with cognitive (Fors et al., 2009; Horvat et al., 2014; Kobrosly et al., 2011; Lyu & Burr, 2016) and physical capability in midlife (Birnie et al., 2011). Similar to our findings, these studies consistently report lower childhood SEP to be associated with poorer cognitive and physical capabilities in adulthood. However, few studies have assessed associations of psychosocial adversity in childhood with cognitive capability later in life (Anda et al., 2006; Feeney et al., 2013; Lovallo et al., 2013; Navalta, Polcari, Webster, Boghossian, & Teicher, 2006; Reuben et al., 2016; Richards & Wadsworth, 2004). Most existing studies have found various types of psychosocial adversity (mainly abuse and neglect) to be associated with poorer cognitive capability in later life (Lovallo et al., 2013; Navalta et al., 2006; Richards & Wadsworth, 2004). Only three studies consider the co-occurrence of multiple forms of psychosocial and/or socioeconomic adversity (Anda et al., 2006; Lovallo et al., 2013; Reuben et al., 2016). All of those studies used a simple additive summary score (i.e., totaled the number of adverse experiences) and found that greater adversity in childhood and adolescence was associated with poorer cognitive outcomes. We are unaware of any studies that have assessed associations of psychosocial adversity in childhood with objectively measured physical capability later in life, only those using self-reported measures of physical capability (Montez & Hayward, 2014; Schussler-Fiorenza Rose, Xie, & Stineman, 2014; Surtees & Wainwright, 2007). These studies reported psychosocial adversity (maltreatment, abuse, and household dysfunction) to be associated with greater risk of physical disability in later life.

### Strengths and Limitations

To the best of our knowledge, this is the first study to assess associations between psychosocial adversity in childhood and objective measures of physical capability in adulthood. Our analytical approach for assessing the effects of total psychosocial adversity improves on existing studies that either assess the relationship between a single type of adversity (because this ignores co-occurrence and likely total effects) or simply add up the number of adverse experiences into a score (because this weights each form of adversity equally). Alternative weighting methods based on theory would also be possible, but it requires making assumptions about the relative severity of each type of adversity for a particular outcome. We had data for a variety of cognitive and physical capability tests, which allowed us to assess the effect of childhood adversity on different aspects of cognition and physical capability and also on overall cognitive and physical functioning.

One limitation of our study is the possibility of selection bias; outcomes were assessed approximately 25 years after recruitment into the cohort. The sample included in this analysis represents approximately 16% of the original ALSPAC mothers’ cohort; thus, as in all longitudinal cohort studies, selection bias because of loss to follow-up is possible. Our study sample also includes a larger proportion of high SEP participants than were initially recruited into ALSPAC. Although this means the prevalence of childhood socioeconomic and psychosocial adversity in our sample may not be representative of the general population, there is evidence that such nongeneralizability often does not result in bias in exposure-outcome associations (Nohr, Frydenberg, Henriksen, & Olsen, 2006). It is also likely that any bias would be toward the null (Howe, Tilling, Galobardes, & Lawlor, 2013), which may, at least in part, explain the lack of an observed association between total psychosocial adversity and cognitive capability.

Psychosocial adversity data were retrospectively self-reported in adulthood, meaning there is potential for recall bias. There is currently no gold standard method for collecting data on adverse experiences in childhood, and a previous review reported retrospective recall in adult life of exposure to adverse experiences in childhood to be sufficiently valid (Hardt & Rutter, 2004). Two existing studies have compared associations of prospectively and retrospectively assessed childhood adversity measures, with various health outcomes in adulthood. The first study (Patten et al., 2015) concluded that associations between childhood adversities and health outcomes during adulthood are not merely artifacts of recall bias and that retrospective and prospective assessment strategies produced very similar results. The second study (Reuben et al., 2016) reported that retrospective and prospective measures of adversity showed moderate agreement (*r* = .47, *p* < .001) and that both associated with all midlife outcomes. They also noted that retrospective childhood adversity measures may be biased toward underestimating the impact of adversity on objectively measured life outcomes.

Despite reports of childhood adversity being retrospectively reported, reverse causation is extremely unlikely in this study. Childhood adversity was retrospectively reported an average of 23 years prior to the assessment of physical and cognitive capability (childhood adversity was retrospectively reported at mean age 29 years, physical and cognitive capability was assessed at mean age 51 years). Thus, we are able to draw some conclusions about the temporality of events because cognitive and physical capability at average age 51 years is extremely unlikely to affect (a) whether participants experienced psychosocial adversity (such as sexual abuse or parental divorce) during childhood and (b) whether participants accurately reported experiencing adversity in childhood, 23 years prior to the cognitive and physical capability assessment. The model fit for the nonsexual abuse factor was slightly poorer than the other models (i.e., root mean square error of approximation and comparative fit index were greater). That said, the factor loadings for all items were relatively high, and modifications to this factor did not substantially improve model fit. Nonsexual abuse has been identified as a potentially important form of psychosocial adversity to consider from a theoretical perspective (Edwards et al., 2003; Lindert et al., 2014; Rich-Edwards et al., 2012); thus, we decided to keep this factor in the analyses despite its slightly lower model fit, particularly given that the overall total psychosocial adversity factor had very good model fit, even with nonsexual abuse included. Our mediation analysis assumes no measurement error in the mediator, and, given our single measure of adult SEP (occupational social class), we are unable to rule this out. Finally, we studied only British women; thus, we cannot assume that our results would generalize to men or women from different ethnic backgrounds. The United Kingdom has low social mobility (Social Mobility Commission, 2016), with women in particular facing challenges in trying to mobilize upward from a low SEP. That said, in our study of women, we do observe social mobility in both directions; 23% of women with low childhood SEP went on to have high adulthood SEP, and 58% of those women with high childhood SEP went on to have a low adulthood SEP.

## Conclusions

In conclusion, our results suggest that lower SEP in childhood is detrimental to both cognitive and physical capability in women in midlife. Greater psychosocial adversity in women is also associated with poorer physical capability, independently of social disadvantage. We found no evidence of an association between psychosocial adversity in childhood and cognitive capability in women, which may, at least in part, be explained by selection bias. Thus, further studies are needed to clarify this association. Our findings suggest that the adverse effects of psychosocial adversity during childhood on objective measures of physical aging in women are independent of social disadvantage in childhood and are also not completely mediated through SEP attained in adulthood. Thus, interventions to both identify and provide support to children experiencing socioeconomic or psychosocial adversity as early as possible, may help to minimize the adverse consequences on cognitive and physical health later in life.

## Supplementary Material

10.1037/pag0000182.supp

## Figures and Tables

**Table 1 tbl1:** Characteristics of Participants Included in the Study (N = 2221), and Excluded Due to Missing Data

Characteristics	Included participants (*N* = 2,221)	Excluded participants^a^		*p* value for difference
	Mean (SD)/median (IQR)	*N* with available data^b^	Mean (SD)/median (IQR)	
Outcomes				
Grip strength, kg^c^	26.1 (6.6)	527	25.8 (7.2)	.32
Chair rise time, s	23.4 (5.4)	479	23.8 (5.8)	.14
Standing balance test with eyes closed, s^d^	4.8 (3.0, 9.9)	506	5.0 (2.9, 9.4)	.82
Three-meter timed walk speed, m/s	1.25 (1.07, 1.36)	522	1.20 (1.07, 1.25)	.01
Logical memory test score^d^	16 (13, 18)	487	15 (13, 17)	<.01
Digit-backward test score^d^	7 (5, 9)	485	7 (5, 9)	.04
Spot-the-word test score^d^	45 (39, 50)	480	44 (37, 49)	.01
Digit symbol coding test score^d^	82 (72, 90)	464	80 (69, 88)	<.01
Verbal fluency test score^d^	43 (35, 51)	464	40 (32, 51)	<.01
Delayed logical memory test score^d^	15 (12, 17)	469	14 (11, 16)	<.01
Covariables				
Age at outcome assessment, years^d^	50.6 (48, 53.6)	550	50.4 (47.6, 53.7)	.65
Ethnicity				
White	98.0%	10,027	97.2%	.04
Nonwhite	2.0%		2.8%	
Father’s (childhood) SEP		8,093		
Nonmanual	57.3%		44.6%	<.001
Manual	42.7%		55.4%	
Adulthood SEP		9,199		
High	68.8%		51.8%	<.001
Low	31.2%		48.2%	
*Note.* SEP = socioeconomic position; IQR = interquartile range.				
^a^ Excluded participants are ALSPAC participants who were missing data for all psychosocial adversity variables, at least one of the physical and cognitive outcomes, and/or potential confounders. ^b^ The *N* with available data column shows the number of excluded participants with data for that particular variable. ^c^ For ease of interpretation of the average values, we present grip strength here. In the analyses, however, we used height-adjusted grip strength. ^d^ Median and interquartile range are presented for nonnormally distributed variables. For continuous variables, the difference between the means of those included and excluded from the analysis was tested using an unpaired *t* test. For categorical variables, the difference between those included and excluded from the analysis was tested using Pearson’s χ^2^ test. For nonnormally distributed variables, differences between those included and excluded from the analysis were tested using a Mann-Whitney *U* test.				

**Table 2 tbl2:** Associations of Childhood SEP With Composite Scores of Cognitive Capability at Mean Age 51 Years (N = 2,221)

Variables	Unadjusted		Adjusted for age at outcome assessment and ethnicity		Adjusted for age at outcome assessment, ethnicity, and cumulative psychosocial adversity		Adjusted for age at outcome assessment, ethnicity, cumulative psychosocial adversity, and adult SEP	
	Standardized β [95% CI]	*p*	Standardized β [95% CI]	*p*	Standardized β [95% CI]	*p*	Standardized β [95% CI]	*p*
Childhood SEP								
Managerial and technical versus professional	−.21 [−.35, −.06]	.005	−.20 [−.34, −.06]	.006	−.20 [−.34, −.06]	.006	−.16 [−.30, −.03]	.02
Skilled nonmanual versus professional	−.28 [−.46, −.13]	.001	−.27 [−.44, −.11]	.001	−.27 [−.44, −.11]	.001	−.19 [−.34, −.03]	.02
Skilled manual versus professional	−.66 [−.80, −.52]	<.001	−.64 [−.78, −.50]	<.001	−.64 [−.78, −.50]	<.001	−.50 [−.64, −.37]	<.001
Partly or unskilled manual versus professional	−.76 [−.95, −.57]	<.001	−.74 [−.92, −.55]	<.001	−.73 [−.92, −.55]	<.001	−.57 [−.75, −.39]	<.001
Covariables								
Age at outcome assessment			.01 [.003, .02]	.01	.01 [.003, .02]	.01	−.001 [−.01, .008]	.76
Ethnicity (nonwhite vs. white)			−.27 [−.55, .02]	.07	−.26 [−.55, .03]	.08	−.18 [−.46, .10]	.163
Cumulative psychosocial adversity					−.01 [−.07, .04]	.63	.005 [−.05, .06]	.86
Adult SEP							−.29 [−.34, −.25]	<.001
*Note.* SEP = socioeconomic position; CI = confidence interval. Standardized beta coefficients are interpreted as a standardised mean difference in the outcome in each childhood SEP group when compared, professional SEP. Results are adjusted for potential confounding by age at outcome assessment, ethnicity, and psychosocial adversity. The final model is adjusted for potential mediation by adult SEP, which is a categorical variable with the same categories as childhood SEP but entered as a linear term (i.e., per category increase in adult SEP).								

**Table 3 tbl3:** Associations of Childhood SEP With Composite Scores of Physical Capability at Mean Age 51 Years (N = 2,221)

Variables	Unadjusted		Adjusted for age at outcome assessment and ethnicity		Adjusted for age at outcome assessment, ethnicity, and cumulative psychosocial adversity		Adjusted for age at outcome assessment, ethnicity, cumulative psychosocial adversity, and adult SEP	
	Standardized β [95% CI]	*p*	Standardized β [95% CI]	*p*	Standardized β [95% CI]	*p*	Standardized β [95% CI]	*p*
Childhood SEP								
Managerial and technical versus professional	−.09 [−.24, .05]	.22	−.11 [−.26, .03]	.13	−.11 [−.26, .03]	.13	−.10 [−.24, .05]	.19
Skilled nonmanual versus professional	−.19 [−.35, −.02]	.03	−.23 [−.39, −.06]	.006	−.23 [−.39, −.07]	.006	−.19 [−.36, −.03]	.02
Skilled manual versus professional	−.26 [−.40, −.11]	.001	−.33 [−.47, −.19]	<.001	−.33 [−.47, −.18]	<.001	−.27 [−.41, −.12]	<.001
Partly or unskilled manual versus professional	−.34 [−.53, −.15]	<.001	−.40 [−.59, −.21]	<.001	−.40 [−.59, −.21]	<.001	−.33 [−.52, −.14]	.001
Covariables								
Age at outcome assessment			−.04 [−.05, −.03]	<.001	−.04 [−.05, −.03]	<.001	−.05 [−.06, −.04]	<.001
Ethnicity (nonwhite vs. white)			−.17 [−.46, .12]	.25	−.15 [−.45, .14]	.31	.12 [−.41, .17]	.43
Cumulative psychosocial adversity					−.04 [−.09, .01]	.15	−.03 [−.08, .02]	.24
Adult SEP							−.13 [−.18, −.08]	<.001
*Note.* SEP = socioeconomic position; CI = confidence interval. Standardized beta coefficients are interpreted as a standardized mean difference in the outcome in each childhood SEP group when compared, professional SEP. Results are adjusted for potential confounding by age at outcome assessment, ethnicity, and psychosocial adversity. The final model is adjusted for potential mediation by adult SEP, which is a categorical variable with the same categories as childhood SEP but entered as a linear term (i.e., per category increase in adult SEP).
