# Supplementary material for: Adversity in Childhood and Measures of Aging in Midlife: Findings From a Cohort of British Women
Source: Psychol Aging. 2017 Sep;32(6):521–30. doi: 10.1037/pag0000182 (PMC5592847; doi:10.1037/pag0000182)
Supplement: Supplementary file 1 [file PAG_Anderson_Supp_Mat.docx]

**Online supplement**

**Assessment of cognitive and physical capability**

***Cognitive tests***

To assess logical memory, a standardised recording of a short story was played to the participant. They were asked to listen carefully and try to remember it the way it was said. After playing the story, the participant was asked to tell the fieldworker everything they could remember about the story, starting at the beginning. One point was scored for each correct item recalled according to standard scoring guidelines. To assess delayed logical memory, the participant was asked to recall the story again after completing all other cognitive tests. To assess backwards digit span, the fieldworker gave a series of numbers out loud and asked the participant to recall them backwards with no time for pause. There were two practise items and seven test items (each item having two trials). Each item had one more number to recall than the preceding item (item 1 had two digits, item 7 had eight digits). The test was discontinued if a participant scored 0 on both trials of any item. In the spot-the-word test the participant was given a series of sixty pairs of words. Each pair contained one real and one nonsense word. The participant was asked to place a tick next to the word in each pair that they thought was the real word. The participant was scored 1 point for each correct answer. In the digit symbol coding test the participant was shown a series of symbols, each one associated with the numbers 1 to 9. They were then asked to fill in a grid drawing the correct symbol next to each number one after the other without skipping any. The task was timed for 120 seconds. The participant was scored 1 point for each correct entry done in the allocated time. To assess verbal fluency, the participant was given a letter of the alphabet (C, F and L) and asked to say as many words as they can think of that beginning with that letter. They were not allowed to include proper nouns or include the same word with a different ending. A practise was given with the letter ‘S’, after which, 1 minute periods were timed for the letters ‘C’, ‘F’ and ‘L’. The participant scored one point for each correct entry done in the allocated time with a total score obtained as the sum of all three scores.

***Physical capability tests***

Two values of hand grip strength were recorded to the nearest 1kg for each hand, using the Jamar handgrip dynamometer. The highest value was used in analyses. Chair rise speed was measured as the time taken (in seconds) for the participant to rise from a sitting to a straight-legged fully standing position, keeping their arms folded, and sit back down 5 times as fast as they could. For the standing balance test, the participant was asked to stand next to a table with arms straight down by their sides (not moved to aid balance). The participant was asked to choose a leg and raise it off the floor to ankle height, bending the leg so the foot is relaxed at ankle level, with their eyes closed. The participant was timed until they lost their balance, dropped their foot or had to reach out to the table for support. If the participant remained on one leg for longer than 30 seconds they were stopped. For the timed 3 metre walk, 3 metres were marked out between two pieces of black tape on the floor. The participant was asked to walk between the two at their usual pace. The time taken was recorded in seconds and the measure was converted to speed in meters per second.

**Rescaling the cognitive and physical capability measures**For cognitive capability, participants with test scores above the 99^th^ percentile were coded to the 99^th^ percentile value. Participant scores were then divided by the 99^th^ percentile value give a value between 0 and 1, with one being equal to the 99^th^ percentile score. For physical capability, grip strength was adjusted for body size by dividing it by height. Height-adjusted grip strength was then divided by the value of the 99th percentile. Participants with values greater than the 99^th^ percentile value were assigned this value. Chair rise time was rescaled as 1 - (time/99^th^ percentile value of time). Participants taking longer than 99^th^ percentile value for time were assigned this value. Standing balance time was rescaled by dividing the total time standing with eyes closed by the maximum possible time (i.e. 30 seconds).The 3-metre timed walk was rescaled as 1 - (time/99^th^ percentile value of time). Participants taking longer than 99^th^ percentile value for time were assigned this.

All rescaled measures were coded in the same direction so that 0 represents the poorest performance and 1 represents highest performance (i.e the 99^th^ percentile). Participants unable to perform each test for health reasons were assigned a value of 0. The four rescaled physical function and six rescaled cognitive capability test scores were summed to create two normally distributed aggregate scores ranging from of 0 to 4, and 0 to 6, respectively.

**Statistical analysis
*Confirmatory factor analyses***

Factor analyses were conducted using Mplus version 7.31 (Muthén & Muthén, 2008). Confirmatory first order factor analyses were used to estimate continuous latent constructs from responses to multiple questions (detailed in Table S1 below) about lack of maternal care, maternal overprotection, sexual abuse, the relationship between their mother and father, childhood physical illness, parental mental illness and absence of the mother or father in the household. (Figure S1) Table S1 details factor loadings for each question asked (i.e. how well each variable loaded onto the factor) with larger numbers representing a better loading. Any value above 0.4 was considered to be an acceptable factor loading, although this value is arbitrary. The majority of variables loaded onto their corresponding first order factors very well. To assess the fit of each first order factor model, three fit statistics were used: (1) root-mean-square error of approximations (RMSEA)(1); (2) comparative fit index (CFI)(2); and (3) Tucker– Lewis fit index (TLI).(3) Table S1 below details the model fit statistics for each factor. The first order factors fit well. The ‘maternal overprotection’ factor had slightly lower CFI and TLI values than the other factors, however, the factor loading of each variable was large and the RMSEA was also reasonable.

***Total psychosocial adversity in childhood***A second order factor analysis model was conducted which included all continuous latent constructs listed above, plus observed binary variables derived from responses to questions about parental disability, parental physical illness, physical abuse, emotional abuse, emotional neglect, physical neglect, parental divorce or separation, death of mother or father in childhood, adoption, and time spent in local authority care as a child (Figure S2). We estimated a latent construct of total psychosocial adversity, whereby adverse experiences that are more strongly correlated with other adverse experiences (and therefore experiences that more likely co-occur) are assigned higher factor loadings, and therefore contribute more to the factor score. Table S1 details the factor loadings for each of the continuous latent constructs and the observed binary variables onto the second order factor. Latent constructs and observed binary variables loaded onto the second order factor well, with the exception of ‘parental death’ and ‘adoption’ which both had factor loadings around 0.2. Excluding these two variables from the second order factor model made no difference to the overall model fit, thus we decided to include them in the final model due to their a priori theoretical importance. As above, RMSEA, CFI and TLI were used to assess model fit (Table S1 below). Model fit was reasonable given the complexity of the second order factor model.

**Table S1. Prevalence and model fit for psychosocial adversity factors (n=2221)**

| **LATENT ADVERSITY CONSTRUCT**  Exact questions asked in questionnaires | **% Prevalence in included*** | **% Prevalence in excluded**** | **P for difference***** | **Factor loadings for each variable** | **Model fit statistics for each latent construct** |
| --- | --- | --- | --- | --- | --- |
| **First order factors** | | | | | |
| **MATERNAL LACK OF CARE** |  |  |  |  |  |
| Did your mother speak to you in a warm and friendly voice? (RS) | 16.6 (361) | 22.1 (2208) | <0.001 | 0.88 | RMSEA = 0.05  CFI =0.98 TLI = 0.97 |
| Did your mother help you as much as you needed? (RS) | 17.0 (371) | 20.4 (2040) | <0.001 | 0.91 |  |
| Did your mother seem emotionally cold to you? | 4.7 (103) | 4.9 (492) | 0.70 | 0.75 |  |
| Problems understood by the mother (RS) | 48.8 (1066) | 49.7 (4985) | 0.45 | 0.82 |  |
| Was your mother affectionate towards you? (RS) | 26.8 (586) | 30.6 (3068) | <0.001 | 0.85 |  |
| Did your mother make you feel you were not wanted? | 2.3 (50) | 4.3 (428) | <0.01 | 0.61 |  |
| Did your mother talk things over with you? (RS) | 56.9 (1243) | 55.4 (5556) | 0.18 | 0.81 |  |
| Did your mother praise you? (RS) | 43.2 (943) | 49.0 (4900) | <0.001 | 0.80 |  |
| Did your mother enjoy talking things over with you? (RS) | 19.5 (417) | 19.6 (1932) | 0.86 | 0.87 |  |
| Did your mother frequently smile at you? (RS) | 9.7 (210) | 10.7 (1065) | 0.18 | 0.91 |  |
| Did your mother seem to understand what you needed or wanted? (RS) | 21.9 (470) | 22.2 (9838) | 0.79 | 0.91 |  |
| Did your mother make you feel better when you were upset? (RS) | 12.5 (268) | 14.0 (9891) | 0.06 | 0.87 |  |
| **MATERNAL OVERPROTECTION** |  |  |  |  |  |
| Did your mother allow you to things you liked doing? (RS) | 36.7 (797) | 43.4 (4360) | <0.01 | 0.65 | RMSEA = 0.07  CFI =0.88 TLI = 0.85 |
| Did your mother try to control what you did? | 23.8 (519) | 25.7 (2577) | 0.06 | 0.53 |  |
| Did your mother let you decide things for yourself? (RS) | 6.3 (137) | 8.5 (853) | 0.001 | 0.75 |  |
| Did your mother give you the freedom you wanted? (RS) | 51.2 (1116) | 55.5 (5568) | <0.001 | 0.86 |  |
| Did your mother let you go out as often as you wanted? (RS) | 58.9 (1285) | 65.4 (6553) | <0.001 | 0.75 |  |
| Was your mother overprotective of you? | 17.7 (385) | 19.8 (1974) | 0.03 | 0.57 |  |
| Did your mother allow you to dress in any way you pleased? (RS) | 14.5 (313) | 15.9 (1576) | 0.10 | 0.68 |  |
| **MALADAPTIVE FAMILY FUNCTIONING** |  |  |  |  |  |
| Was your parent's behaviour stable and predictable to you as a child? (RS) | 9.7 (212) | 13.1 (1318) | <0.001 | 0.76 | RMSEA = 0.06  CFI =0.98 TLI = 0.97 |
| Did your parents have serious arguments? | 26.5 (584) | 26.9 (2707) | 0.69 | 0.82 |  |
| Was your parent’s relationship violent? | 9.6 (192) | 14.6 (1000) | <0.001 | 0.89 |  |
| Was your parent’s relationship affectionate? (RS) | 12.2 (246) | 12.6 (870) | 0.66 | 0.81 |  |
| Was your parent’s relationship quarrelsome? | 74.4 (1505) | 78.6 (5485) | <0.001 | 0.62 |  |
| Was your parent’s relationship happy? (RS) | 4.2 (85) | 5.4 (375) | 0.04 | 0.95 |  |
| Was your parent’s relationship frightening? | 17.5 (350) | 19.7 (1356) | 0.03 | 0.84 |  |
| Was your parent’s relationship friendly? (RS) | 2.9 (58) | 3.9 (272) | 0.03 | 0.93 |  |
| Was your parent’s relationship respectful? (RS) | 6.8 (138) | 9.5 (655) | <0.001 | 0.89 |  |
| Was your parent’s relationship remote or distant? | 42.1 (844) | 42.2 (2898) | 0.93 | 0.74 |  |
| Parental separation | 10.9 (240) | 18.4 (1856) | <0.001 | 0.70 |  |
| **PARENTAL MENTAL ILLNESS** |  |  |  |  |  |
| Was either parent mentally ill before age 17 years? | 4.4 (98) | 4.2 (419) | 0.55 | 0.90 | RMSEA = 0.03  CFI =0.94 TLI = 0.91 |
| Did your mother have depression or nerves? | 20.1 (422) | 22.3 (2143) | 0.03 | 0.52 |  |
| Did your mother have an alcohol problem? | 2.2 (48) | 2.8 (280) | 0.12 | 0.69 |  |
| Did your father have an alcohol problem? | 4.7 (101) | 6.8 (659) | <0.01 | 0.39 |  |
| **SEXUAL ABUSE** |  |  |  |  |  |
| Unwanted or abusive masturbation^a^ | 3.7 (75) | 4.1 (368) | 0.31 | 0.70 | RMSEA = 0.02  CFI =0.98 TLI = 0.97 |
| Unwanted or abusive fondling^a^ | 9.9 (207) | 10.0 (903) | 0.92 | 0.85 |  |
| Unwanted or abusive genital rubbing^a^ | 5.1 (107) | 6.0 (545) | 0.11 | 0.89 |  |
| Unwanted or abusive oral sex^a^ | 1.0 (20) | 2.1 (186) | <0.001 | 0.85 |  |
| Unwanted or abusive intercourse^a^ | 1.1 (25) | 2.4 (215) | <0.001 | 0.84 |  |
| **NON-SEXUAL ABUSE** |  |  |  |  |  |
| Parent was physically cruel | 2.4 (53) | 3.7 (368) | <0.01 | 0.82 | RMSEA = 0.12  CFI =0.89 TLI = 0.73 |
| Parent was emotionally cruel | 7.3 (160) | 7.7 (778) | 0.45 | 0.97 |  |
| Physically neglected by parent | 1.0 (22) | 2.2 (168) | <0.001 | 0.73 |  |
| Emotionally neglected by parent | 18.5 (390) | 22.8 (1706) | <0.001 | 0.69 |  |
| **Second order factor** | | | | | |
| **TOTAL PSYCHOSICIAL ADVERSITY** |  |  |  |  | RMSEA = 0.04  CFI =0.94 TLI =0.94 |
| Lack of care factor | - | - | - | 0.79 |  |
| Overprotection factor | - | - | - | 0.53 |  |
| Maladaptive family functioning factor | - | - | - | 0.68 |  |
| Parental mental illness factor | - | - | - | 0.62 |  |
| Sexual abuse factor | - | - | - | 0.39 |  |
| Non-sexual abuse factor | - | - | - | 0.99 |  |

RS=Reverse Score. All prevalence estimates are given for the ‘Yes’ category, except when RS is indicated, where prevalence is given for the ‘No’ category.
^a^Multiple questions were asked about sexual experiences (including unwanted experiences) before age 16. Questions were asked in relation to the type of experience, who was involved in the experience, whether or not the participant wanted it to happen, how old they were when it first happened and how often it happened, and from responses to these questions the abuse variables were derived by the ALSPAC data team.
*Prevalence of exposure in participants included in the analysis
**Prevalence of exposure in participants excluded from the analysis due to missing data for the adversity exposures, the outcomes or the potential confounders and mediators
***P values for differences in prevalence estimates between included and excluded participants were obtained from a chi-squared test.

**Creating the additive score**The additive score was created for participants with complete data for all the adversity measures, and physical and cognitive outcome data (n=1,535). The first step of creating the additive score was to create binary indicators for each type of adversity. Maternal lack of care and maternal overprotection were both continuous scores from a validated index of maternal bonding. We used the methods by Parker and colleagues(4) to create a dichotomous variable of “neglectful parenting” (low care and high protection) versus all other categories. For mothers, scores of 27.0 and 13.5 are used as cut offs for lack of care and overprotection, respectively. Maladaptive family function was captured by 11 binary variables (see Table S1 above). Thus, we created a score from these 11 variables which indicated the number of maladaptive family function exposures. This score was then divided into quintiles, and a binary variable representing maladaptive family functioning was created to indicate those participants in the fifth quintile (i.e. the highest number of maladaptive family function exposures) compared to all other quintiles. Sexual abuse was captured by 5 different questions. A binary variable was created to indicate those participants who answered ‘yes’ to any of the sexual abuse questions. Non-sexual abuse was captured by 4 different questions about physical and emotional abuse or neglect, and a binary variable was created to indicate any participant who answered ‘yes’ to any of those 4 questions. A binary variable was also created to indicate any participant who reported any form of parental mental illness.

Once binary variables were created for each adversity exposure, a score was created by adding up each exposure for each participant. Thus, each participant could have a score between 0 and 5. Table S2 below shows the prevalence of each binary adversity variable included in the score, and the mean (standard deviation) psychosocial adversity score.

**Table S2: Prevalence of each binary psychosocial adversity measure and distribution of the additive psychosocial adversity score**

|  | **Prevalence (Total N=1535)** |
| --- | --- |
| **Neglectful Parenting** | 16.4% |
| **Maladaptive Family Functioning** | 18.3% |
| **Parental Mental Illness** | 29.4% |
| **Sexual Abuse** | 12.1% |
| **Non-Sexual Abuse** | 18.6 |
|  | **Mean (Standard Deviations)** |
| **Total psychosocial adversity** | 0.95 (1.14) |

**Table S3: Pearson’s correlation coefficients of physical capability measures**

|  | **Height-adjusted grip strength** | **Chair rise speed** | **Standing balance test** | **3-metre timed walk speed** |
| --- | --- | --- | --- | --- |
| **Height-adjusted grip strength** | 1.00 |  |  |  |
| **Chair rise speed** | 0.17 | 1.00 |  |  |
| **Standing balance test** | 0.08 | 0.09 | 1.00 |  |
| **3-metre timed walk speed** | 0.07 | 0.25 | 0.07 | 1.00 |

All variables are scaled in the same direction so that higher values reflect better performance

**Table S4: Pearson’s correlation coefficients of cognitive capability measures**

|  | **Verbal fluency test** | **Logical memory test** | **Digit backwards test** | **Digit symbol coding test** | **Delayed logical memory test** | **Spot the word test** |
| --- | --- | --- | --- | --- | --- | --- |
| **Verbal fluency test** | 1.00 |  |  |  |  |  |
| **Logical memory test** | 0.23 | 1.00 |  |  |  |  |
| **Digit backwards test** | 0.35 | 0.24 | 1.00 |  |  |  |
| **Digit symbol coding test** | 0.25 | 0.16 | 0.23 | 1.00 |  |  |
| **Delayed logical memory test** | 0.23 | 0.84 | 0.22 | 0.18 | 1.00 |  |
| **Spot the word test** | 0.41 | 0.33 | 0.33 | 0.15 | 0.33 | 1.00 |

All variables are scaled in the same direction so that higher values reflect better performance

**Table S5. Distribution of adverse psychosocial experiences by childhood SEP (n=2221)**

| **Continuous factor variables** | **Non-manual childhood SEP (n=1272)** | **Manual childhood SEP (n=949)** | **P for difference** |
| --- | --- | --- | --- |
| Sexual abuse | 0.15 (0.53) | 0.16 (0.55) | 0.60 |
| Non-sexual abuse | 0.13 (0.58) | 0.14 (0.59) | 0.79 |
| Parental mental illness | 0.09 (0.59) | 0.10 (0.58) | 0.78 |
| Maladaptive family function | 0.07 (0.77) | 0.14 (0.81) | 0.05 |
| Maternal lack of care | 0.08 (0.81) | 0.11 (0.85) | 0.29 |
| Maternal overprotection | 0.05 (0.81) | 0.03 (0.85) | 0.70 |
| Total psychosocial adversity | 0.12 (0.76) | 0.16 (0.78) | 0.17 |

SEP – socioeconomic position. Distribution in the continuous factor variables is mean (SD) of the factor scores and P for difference was obtained using a two-tailed t-test. Distribution of binary observed variables is presented as a percent prevalence in the exposed group and the P for difference was obtained from a chi^2^ test.

**Table S6. Associations of total psychosocial adversity with composite scores of cognitive capability at mean age 51 years (N=2221)**

|  | **Unadjusted** |  | **Adjusted for age at outcome assessment and ethnicity** | | **Adjusted for age at outcome assessment, ethnicity and childhood SEP** | | **Adjusted for age at outcome assessment, ethnicity, childhood SEP and adult SEP** | |
| --- | --- | --- | --- | --- | --- | --- | --- | --- |
|  | **Standardised β (95% CI)** | **P** | **Standardised β (95% CI)** | **P** | **Standardised β (95% CI)** | **P** | **Standardised β  (95% CI)** | **P** |
| **Total psychosocial adversity** | **-0.04 (-0.09, 0.01)** | **0.16** | **-0.04 (-0.09, 0.01)** | **0.12** | **-0.02 (-0.07, 0.03)** | **0.37** | **-0.004 (-0.05, 0.05)** | **0.86** |
| Covariables |  |  |  |  |  |  |  |  |
| Age at outcome assessment |  |  | 0.09 (0.05, 0.13) | <0.001 | 0.06 (0.02, 0.10) | 0.01 | -0.004 (-0.04, 0.04) | 0.83 |
|  |  |  |  |  |  |  |  |  |
| Ethnicity (non-white vs white) |  |  | -0.04 (-0.07, 0.004) | 0.03 | -0.24 (-0.28, -0.20) | <0.001 | -0.03 (-0.06, 0.01) | 0.12 |
|  |  |  |  |  |  |  |  |  |
| Childhood SEP |  |  |  |  | -0.04 (-0.07, 0.003) | 0.04 | -0.19 (-0.23, -0.15) | <0.001 |
|  |  |  |  |  |  |  |  |  |
| Adult SEP |  |  |  |  |  |  | -0.27 (-0.31, -0.23) | <0.001 |

SEP – socioeconomic position. CI – confidence interval.
Standardised beta coefficients are interpreted as a standard deviation increase in cognitive function per one standard deviation increase in total psychosocial adversity. Results are adjusted for potential confounding by age at outcome assessment, ethnicity and Childhood SEP. The final model is adjusted for potential mediation by adult SEP. Childhood and adulthood SEP are categorical variables with the categories ‘professional’, ‘managerial/technical’, ‘skilled non-manual’, ‘skilled manual’, and ‘partly or unskilled manual’ and are entered as linear terms (i.e. per category increase in childhood and adulthood SEP).

**Table S7. Associations of total psychosocial adversity with composite scores of physical capability at mean age 51 years (N=2221)**

|  | **Unadjusted** |  | **Adjusted for age at outcome assessment and ethnicity** | | **Adjusted for age at outcome assessment, ethnicity and childhood SEP** | | **Adjusted for age at outcome assessment, ethnicity, childhood SEP and adult SEP** | |
| --- | --- | --- | --- | --- | --- | --- | --- | --- |
|  | **Standardised β (95% CI)** | **P** | **Standardised β (95% CI)** | **P** | **Standardised β (95% CI)** | **P** | **Standardised β (95% CI)** | **P** |
| **Total psychosocial adversity** | **-0.07 (-0.12, -0.02)** | **0.01** | **-0.05 (-0.10, 0.0004)** | **0.05** | **-0.04 (-0.09, 0.01)** | **0.10** | **-0.03 (-0.08, 0.02)** | **0.18** |
| Covariables |  |  |  |  |  |  |  |  |
| Age at outcome assessment |  |  | -0.16 (-0.20, -0.12) | 0.00 | -0.18 (-0.22, -0.14) | 0.00 | -0.2 (-0.25, -0.16) | 0.00 |
|  |  |  |  |  |  |  |  |  |
| Ethnicity (non-white vs white) |  |  | -0.02 (-0.06, 0.02) | 0.26 | -0.02 (-0.05, 0.02) | 0.30 | -0.10 (-0.14, -0.06) | 0.00 |
|  |  |  |  |  |  |  |  |  |
| Childhood SEP |  |  |  |  | -0.12 (-0.16, -0.08) | 0.00 | -0.10 (-0.14, -0.06) | 0.00 |
|  |  |  |  |  |  |  |  |  |
| Adult SEP |  |  |  |  |  |  | -0.12 (-0.16, -0.07) | 0.00 |

SEP – socioeconomic position. CI – confidence interval.
Standardised beta coefficients are interpreted as a standard deviation increase in cognitive function per one standard deviation increase in total psychosocial adversity. Results are adjusted for potential confounding by age at outcome assessment, ethnicity and Childhood SEP. The final model is adjusted for potential mediation by adult SEP. Childhood and adulthood SEP are categorical variables with the categories ‘professional’, ‘managerial/technical’, ‘skilled non-manual’, ‘skilled manual’, and ‘partly or unskilled manual’ and are entered as linear terms (i.e. per category increase in childhood and adulthood SEP).

**Table S8. Associations of individual types of psychosocial adversity with composite scores of cognitive and physical capability at mean age 51 years (N=2221)**

|  | **Cognitive function** | |  | **Physical function** | |
| --- | --- | --- | --- | --- | --- |
| **Continuous variables** | **Mean difference (95% CI)** | **P** |  | **Mean difference (95% CI)** | **P** |
| Sexual abuse | 0.04 (-0.03, 0.11) | 0.28 |  | 0.01 (-0.06, 0.08) | 0.84 |
| Non-sexual abuse | -0.04 (-0.1, 0.03) | 0.31 |  | -0.04 (-0.11, 0.02) | 0.20 |
| Maladaptive family function | 0.02 (-0.04, 0.07) | 0.56 |  | -0.01 (-0.06, 0.04) | 0.70 |
| Parental mental illness | 0.05 (-0.02, 0.12) | 0.21 |  | -0.01 (-0.07, 0.06) | 0.88 |
| Maternal lack of care | -0.03 (-0.08, 0.01) | 0.18 |  | -0.05 (-0.09, 0.001) | 0.05 |
| Maternal overprotection | -0.06 (-0.10, -0.01) | 0.01 |  | -0.04 (-0.08, 0.01) | 0.11 |

SEP – socioeconomic position. Continuous exposure variables are latent factor scores derived from multiple variables related to the overall construct (see supplementary material for details); beta coefficients are interpreted as the average change in standard deviations of the outcome per standard deviation higher factor score. Binary exposure variables are observed variables and coefficients are interpreted as the standardised mean difference in the outcome between the exposed group and the unexposed group. Coefficients are adjusted for age at outcome assessment, ethnicity and childhood SEP.

**Table S9. Associations of SEP and psychosocial adversity in childhood with individual cognitive and physical capability measures (N=2221). All measures are scaled in the same direction for comparability.**

|  | **SEP in childhood** | |  | **Psychosocial adversity in childhood** | |
| --- | --- | --- | --- | --- | --- |
| **Cognitive capability tests** | **Mean difference (95% CI)** | **P** |  | **Mean difference (95% CI)** | **P** |
| Logical memory test score (SD) | -0.15 (-0.27, -0.03) | 0.01 |  | -0.01 (-0.06, 0.04) | 0.72 |
| Digit backwards test score (SD) | -0.14 (-0.26, -0.03) | 0.02 |  | 0.01 (-0.04, 0.06) | 0.68 |
| Spot the word test score (SD) | -0.24 (-0.39, -0.08) | 0.003 |  | 0.04 (-0.01, 0.09) | 0.09 |
| Digit symbol coding test score (SD) | -0.08 (-0.15, -0.01) | 0.03 |  | -0.09 (-0.14, -0.04) | 0.00 |
| Verbal fluency test score (SD) | -0.14 (-0.26, -0.03) | 0.01 |  | -0.02 (-0.07, 0.03) | 0.35 |
| Delayed logical memory test score (SD) | -0.15 (-0.27, -0.03) | 0.01 |  | -0.03 (-0.09, 0.02) | 0.21 |
| **Physical capability tests** | **Mean difference (95% CI)** | **P** |  | **Mean difference (95% CI)** | **P** |
| Height-adjusted grip strength (SD) | -0.06 (-0.12, 0) | 0.05 |  | -0.04 (-0.09, 0.01) | 0.10 |
| Chair rise (SD) | -0.06 (-0.11, 0) | 0.07 |  | -0.03 (-0.08, 0.02) | 0.24 |
| Standing balance test with eyes closed (SD) | -0.09 (-0.17, -0.01) | 0.03 |  | 0.01 (-0.04, 0.06) | 0.73 |
| 3-metre timed walk (SD) | -0.02 (-0.07, 0.02) | 0.29 |  | -0.08 (-0.13, -0.03) | 0.00 |

SEP – socioeconomic position. Beta coefficients for SEP in childhood are interpreted as a mean difference in standard deviations of the outcome for manual childhood SEP compared to non-manual childhood SEP. Beta coefficients for psychosocial adversity in childhood are interpreted as the average change in standard deviations of the outcome per standard deviation higher total psychosocial adversity score. Models for childhood SEP and psychosocial adversity are mutually adjusted for each other plus age at outcome assessment and ethnicity.

**Table S10. Associations of psychosocial adversity in childhood with composite scores of cognitive and physical capability at mean age 51 years by high and low childhood SEP (N=2221)**

|  | **High Adult SEP (n=1528)** |  | **Low adult SEP (n=693)** |  |  |
| --- | --- | --- | --- | --- | --- |
|  | **Mean difference (95% CI)** |  | **Mean difference (95% CI)** |  | **Interaction P value** |
| **Cognitive capability score (SD)** | -0.02 (-0.09 to 0.05) |  | -0.01 (-0.09 to 0.07) |  | 0.89 |
| **Physical capability score (SD)** | -0.05 (-0.12 to 0.03) |  | -0.04 (-0.11 to 0.04) |  | 0.88 |

SEP – socioeconomic position. Beta coefficients are interpreted as a standard deviation change in the outcome per standard deviation higher total psychosocial adversity score. Results are adjusted for childhood SEP, age at outcome assessment and ethnicity.

**Table S11. Associations of psychosocial adversity in childhood with composite scores of cognitive and physical capability at mean age 51 years by high and low adult SEP (N=2221)**

|  | **High Adult SEP (n=1528)** |  | **Low adult SEP (n=693)** |  |  |
| --- | --- | --- | --- | --- | --- |
|  | **Mean difference (95% CI)** |  | **Mean difference (95% CI)** |  | **Interaction P value** |
| **Cognitive capability score (SD)** | 0.02 (-0.09, 0.10) |  | -0.04 (-0.25, 0.17) |  | 0.34 |
| **Physical capability score (SD)** | -0.02 (-0.16, 0.12) |  | -0.05 (-0.28, 0.19) |  | 0.61 |

SEP – socioeconomic position. Beta coefficients are interpreted as a standard deviation change in the outcome per standard deviation higher total psychosocial adversity score. Results are adjusted for childhood SEP, age at outcome assessment and ethnicity.

**Table S12. Complete case associations of SEP and psychosocial adversity in childhood with composite scores of cognitive and physical capability at mean age 51 years (N=1422)**

|  | **Socioeconomic adversity** | |  | **Psychosocial adversity** | |
| --- | --- | --- | --- | --- | --- |
|  | **B (95% CI)** | **P** |  | **B (95% CI)** | **P** |
| **Cognitive capability score (SD)** | -0.04 (-0.1, 0.02) | 0.17 |  | -0.04 (-0.1, 0.02) | 0.21 |
| **Physical capability score (SD)** | -0.08 (-0.14, -0.02) | 0.01 |  | -0.08 (-0.13, -0.02) | 0.01 |

SEP – socioeconomic position. Results are mutually adjusted for each other plus ethnicity and age at outcome assessment

**Table S13. Associations of an additive score of psychosocial adversity in childhood with composite scores of cognitive and physical capability at mean age 51 years (N=1535)**

|  |  | **Psychosocial adversity** | |
| --- | --- | --- | --- |
|  |  | **B (95% CI)** | **P** |
| **Cognitive capability score (SD)** |  | -0.02 (-0.06, 0.03) | 0.48 |
| **Physical capability score (SD)** |  | -0.03 (-0.07, 0.02) | 0.21 |

Results are adjusted for ethnicity and age at outcome assessment

Figure S1 – Diagram of an example second order factor model


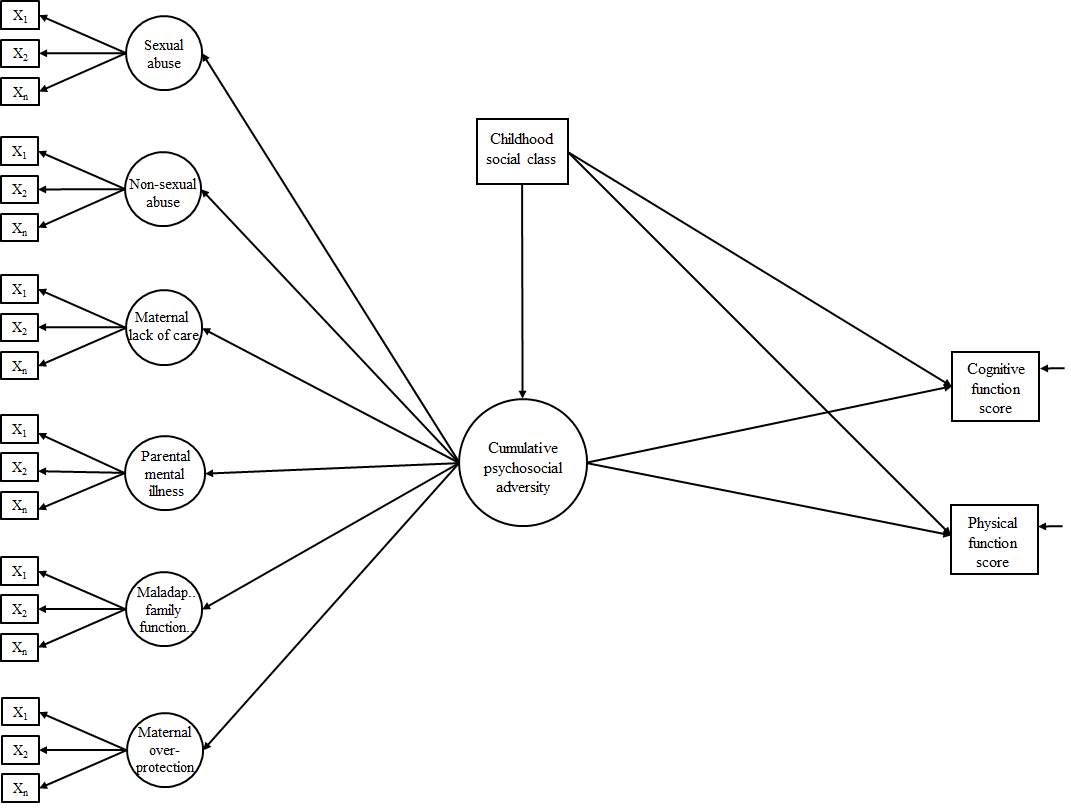


X_1-n_ represent observed binary indicators that load onto each individual latent psychosocial adversity construct (i.e. the first order factors). A complete list of the binary indicators for each latent construct is provided in Table S1, along with their factor loadings and model fit statistics. Total psychosocial adversity is a second order factor that captures each of the individual latent psychosocial adversity constructs. Table S1 provides factor loadings for each of the first order factors onto the second order factor. Childhood social class has been shown as a covariate for illustrative purposes; in our confounder adjusted model, ethnicity and age at outcome assessment were additionally adjusted for. Cognitive and physical function scores are multivariate outcomes with residual covariances.

1. Steiger JL, JC. Statistically based tests for the number of common factors. Annual Spring Meeting of the Psychometric Society; Iowa City, IA1980.

2. Bentler PM. Comparative fit indexes in structural models. Psychol Bull. 1990;107(2):238-46.

3. Tucker LL, C. A reliability coefficient for maximum likelihood factor analysis. Psychometrika. 1973;38(1):1-10.

4. Parker G. The Parental Bonding Instrument. A decade of research. Soc Psychiatry Psychiatr Epidemiol. 1990;25(6):281-2.
